# The impact of operational factors on degradation of formaldehyde as a human carcinogen using Ag_3_ PO_4_ /TiO_2_ photocatalyst

**DOI:** 10.34172/hpp.2023.06

**Published:** 2023-04-30

**Authors:** Asghar Hadi, Aligholi Niaei, Azam Seifi, Yahya Rasoulzadeh

**Affiliations:** ^1^Department of Occupational Health Engineering, Faculty of Health, Tabriz University of Medical Sciences, Tabriz, Iran; ^2^Catalyst & Reactor Research Lab, Department of Chemical & Petroleum Engineering, University of Tabriz, Tabriz, Iran; ^3^Department of Applied Chemistry, Faculty of Chemistry, University of Tabriz, Tabriz, Iran; ^4^Department of Chemistry, Gebze Technical University, Gebze, Kocaeli, Turkey; ^5^Road Traffic Injury Research Center, Tabriz University of Medical Sciences, Tabriz, Iran

**Keywords:** Ag_3_ PO_4_ /TiO_2_, Air, Dip-coating, Formaldehyde, Photocatalytic, Workplace

## Abstract

**Background:** The International Agency for Research on Cancer (IARC) identified formaldehyde as a carcinogen in 2004, yet formaldehyde is widely used in health care settings and various industries. In recent years, photocatalytic oxidation has been developed as a potential technique for removing pollutants arising from organic chemical agents and consequently promoting the health indices. This study investigated the effect of operational factors in optimizing formaldehyde removal from the air using Ag_3_ PO_4_ /TiO_2_ photocatalyst.

**Methods:** An experimental study was designed to investigate the effect of operational factors on the efficiency of formaldehyde degradation. The variables investigated in this study include pollutant retention time, initial pollutant concentration and relative humidity. Sol-gel method was used to synthesize the nano-composite photocatalyst. An ideal experimental design was carried out based on Box-Behnken design (BBD) with response surface methodology (RSM). The sample size in this study includes all the glasses coated with Ag_3_ PO_4_ /TiO_2_ photocatalyst.

**Results:** The maximum formaldehyde degradation of 32% was obtained at the initial concentration of 2 ppm, 20% relative humidity, and 90 minutes of retention time. Based on the statistical results, the correlation coefficient of the present study for the impact of operational factors on formaldehyde degradation was 0.9635, which means that there is only 3.65% probability of error in the model.

**Conclusion:** The operational factors examined in this study (retention time, relative humidity, and initial formaldehyde concentration) were significantly influential in the degradation efficiency of formaldehyde by the photocatalyst. Due to the high exposure of employees and clients of health and treatment centers to formaldehyde as a carcinogenic substance, the results of this study can be used in ventilation systems to remove environmental pollutants in health care centers and other occupational settings.

## Introduction

 The rapid population development and industrialization have increased chemical contaminants.^1^ Volatile organic compounds (VOCs) are recognized as one of the most important chemical groups threatening human health among all hazardous chemicals that reduce air quality. They are considered as a large group of contaminants with a low boiling point. Benzene, formaldehyde, toluene, styrene, xylene, acetaldehyde, naphthalene, and hexanal are among the most important VOCs in indoor environments.^2^

 Formaldehyde is one of the most well-known VOCs due to its highly toxic nature and widespread indoor distribution. Due to its high solubility in water, it is rapidly absorbed through the gastrointestinal and respiratory tracts. Formaldehyde was recognized as a human carcinogen by the IARC in 2004.^3,4^ Also, it is a raw chemical that is frequently employed as a disinfectant and preservative in various industries.^5^ Additionally, it is one of the primary components of many building materials such as wallpaper, wood, and paint, and it may even be produced in activities such as cooking and using electrical appliances.^3,6^ Pathologists, research and medical laboratory staffs are constantly exposed to gaseous formaldehyde because they use it as a tissue stabilizer, disinfectant, and preservative.^7,8^

 According to the study of Soltanpour et al, in both industrial and health care center settings, concentrations of gaseous formaldehyde were higher than the 8-hour threshold limit value - time-weighted average (8-hr TLV-TWA) recommended by the American Conference of Governmental Industrial Hygienists (ACGIH).^9^ Therefore, controlling and reducing occupational exposure to this pollutant in health care centers and industrial sectors is necessary.

 In recent years, photocatalytic oxidation using semiconductors that are activated in visible light has been proposed as a potential technique for removing pollutants arising from organic chemical agents.^10-12^ Photocatalytic degradation, known as one of the advanced oxidation processes, consists of three components: light, semiconductor photocatalyst, and oxygen, which is used to produce free radicals. The photocatalytic process begins with the absorption of light by the photocatalyst. It continues with a series of redox reactions that degrade pollutants, but the photocatalyst remains unchanged in these reactions.^13^

 With a 2.58 eV bandgap, the Ag_3_PO_4_ photocatalyst is one of the most extensively utilized photocatalysts in visible light. However, due to the recombination of the hole and excited electron, this photocatalyst cannot be used in practice.^14^ Heterostructure photocatalysts can overcome the limitations of single-component photocatalysts, such as electron and hole recombination. In this way, the electrons produced in the conduction band and the holes created in the valence band of one photocatalyst are transferred to the conduction band or valence band of the other photocatalyst. Under these conditions, the produced electrons and holes are spatially separated, and the possibility of recombination is reduced.^15-18^ Previous studies have shown that combining Ag_3_PO_4_ with TiO_2_ can increase the performance of Ag_3_PO_4_ up to 12 times.^19^

 Operating factors (such as retention time, pollutant concentration, and relative humidity) can affect the photocatalyst degradation efficiency.^20-23^ The studies conducted in this field have investigated the effect of one factor on degradation efficiency, as a result, the mutual effect of operating factors on degradation efficiency has not been determined. Therefore, with a better understanding of the impact of operational factors on degradation efficiency, this study aimed to evaluate and optimize the impact of these operating factors on degradation efficiency of photocatalyst.

## Materials and Methods

###  Materials

*Titanium tetraisopropoxide (*C_12_H_28_O_4_Ti*, Merck), *silver nitrate (AgNO_3_*, Samchun*), sodium dihydrogen phosphate (NaH_2_PO_4_*, Merck*), nitric acid 65% (HNO_3_, Merck), ethanol 99.5%(C_2_H_5_OH, Merck), acetylacetone (CH_3_COCH_2_COCH_3_, Merck), paraformaldehyde ((CH_2_O)n, Merck ) and acetone (C_3_H_6_O, Merck) were used as purchased without further purifications. A commercial Pyrex glass with a diameter of 8 cm was used to coat the photocatalyst.

###  Synthesis

 To prepare Ag_3_PO_4_, 1.53 g silver nitrate and 0.995 g sodium dihydrogen phosphate were separately dissolved in 25 mL distilled water. The prepared sodium dihydrogen phosphate solution was added dropwise to the silver nitrate solution and stirred at room temperature for 4 hours. The resulting Ag_3_PO_4_ was filtered off, washed with equal volumes of ethanol and water, and dried in an oven at 60°C for 24 hours.

 TiO_2_ particles were prepared by sol-gel method. The initial sol was prepared by mixing titanium isopropoxide (TTIP), acetylacetone (AcAc), and ethanol (EtOH). Finally, nitric acid was added dropwise to obtain a clear solution (The molar ratio of TTIP: AcAc: EtOH: HNO_3_ was 1:1:40:0.1). After two hours of mixing, which is known as aging time, the prepared Ag_3_PO_4_ was added to the solution. Before coating the films on the glass slides, they were first immersed in an ultrasonic bath containing equal volumes of ethanol and acetone for 20 minutes, followed by being immersed in 1% hydrochloric acid solution for 20 minutes. Finally, they were washed with distilled water and placed in an oven at 60°C to dry. In each coating process, the glass substrate was immersed in the prepared sol at a speed of 5 mm/s, and after 10 seconds, it was withdrawn from the solution at the same. Then it was placed in a furnace for 2 h, and at 550 °C for calcination step.

###  Characterization

 The scanning electron microscopy (SEM) images and energy dispersive X-ray analysis spectroscopy were measured by a MIRA3 FEG-SEM field emission scanning electron microscope with an acceleration voltage of 30.0 kV. The UV–Vis diffuse reflectance spectra of the samples were recorded on a Analytik Jena, Specord 250 UV–Vis spectrophotometer in the wavelength range of 300–850 nm.

###  Photocatalytic activity

 A 3-L photoreactor made of stainless steel was used to test the degradation efficiency of the synthesized photocatalyst. After placing the photocatalyst-coated glass in the photoreactor, in each experiment, first, the desired relative humidity was created by evaporating distilled water and transferring it to the photoreactor. Then the desired formaldehyde concentration was generated by sublimation of paraformaldehyde from the photoreactor’s paraformaldehyde tank. The irradiation of the photocatalyst by light was then started and lasted for 30, 60, or 90 minutes through the quartz window of the photoreactor. A 150-W xenon lamp with a cut-off filter of 420 nm was used as the light source, which was placed on top of the photoreactor, 10 cm away from the photocatalyst. Measurement of formaldehyde concentration inside the photoreactor was performed using the acetylacetone method.^24^ The formaldehyde degradation efficiency was calculated according to the following formula:

$$Degradation\;efficiency(\%)\;=\;\frac{C_{0}\;-\;C_{t}\;}{C_{0}}\;\times \;100$$

Eq. 1

 where *C*_0_ is referred to the initial concentration of formaldehyde, and *C*_t_ is the concentration of formaldehyde at the time *t*.

###  Experimental design

 Parameters that investigated for photocatalytical degradation of formaldehyde were analyzed by standard response surface methodology (RSM) Box-Behnken design (BBD). In this method, each variable is evaluated at 3 levels. The levels of study variables are given in Table 1. Design-Expert software version 12 was utilized for this purpose. RSM is a technique for optimizing process parameters to obtain optimum efficiency with the fewest possible tests. According to the BBD method, 12 experiments with different conditions should be performed to investigate the effect of 3 influential parameters, initial formaldehyde concentration, retention time, and relative humidity, at three levels on the percentage of formaldehyde degradation. These 12 experiments were enhanced with five replications to assess the pure error. As a result, a total of 17 experiments were performed.

**Table 1:** Independent parameters and their coded and actual values

**Parameters**	**Unit**	**Symbol**	**Ranges and levels**		
			**-1**	**0**	**+1**
Retention time	min	A	30	60	90
Relative humidity	%	B	20	40	60
Initial formaldehyde concentration	ppm	C	2	3	4

## Results

###  Characterization results

**Figure 1:**
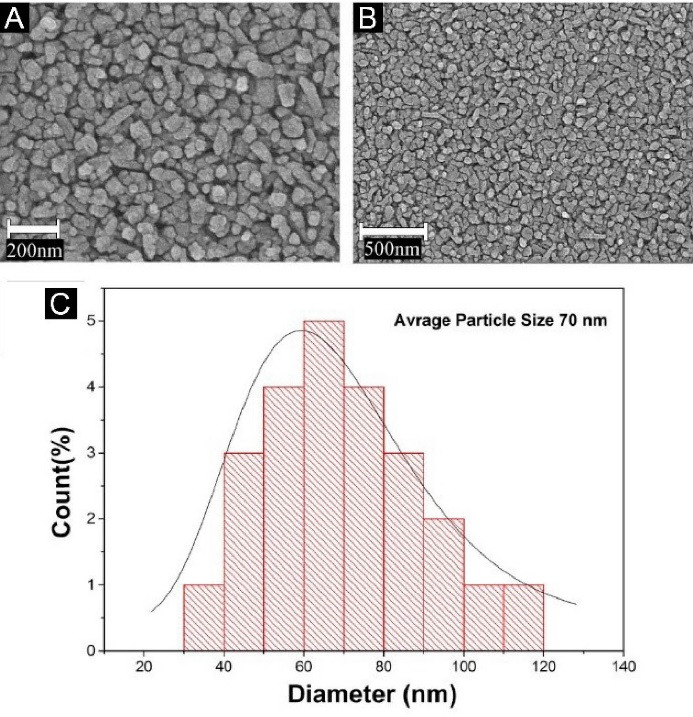
(A) and (B) SEM image of surface of the glass coated with nano-photocatalyst Ag3PO4/TiO2 (C) Coated particle size distribution

 The morphological properties of the produced photocatalyst were evaluated using SEM images, shown in Figure 1A and 1B. As can be seen, the average particle size of the synthesized photocatalyst was 70 nm. Figure 1C depicts the particle size distribution.

 The energy-dispersive X-ray spectroscopy (EDS) of this SEM image confirms the presence of Ti, O, Ag, and P elements. Additionally, element mapping demonstrates that all elements (Ag, P, O, and Ti) are dispersed uniformly.

**Figure 2:**
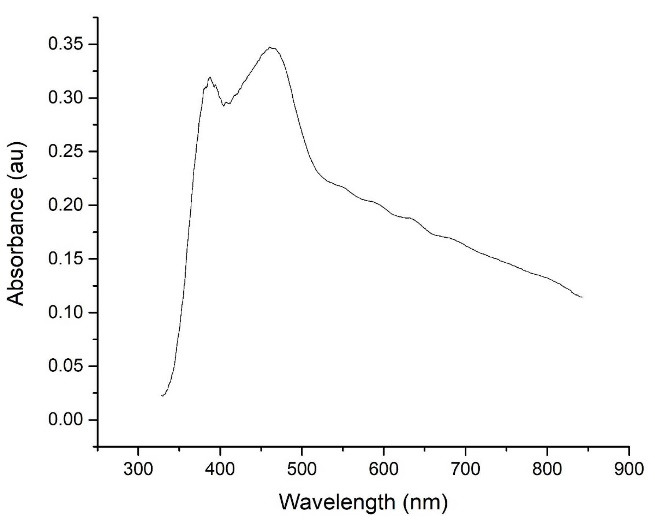
UV–visible absorption spectrum of Ag_3_PO_4_/TiO_2_

 The absorption spectra of the Ag_3_PO_4_/TiO_2_ composite is shown in Figure 2. According to the obtained results, the absorption spectrum of the Ag_3_PO_4_/TiO_2_ composite was a combination of the absorption spectra of these two components (TiO_2_ and Ag_3_PO_4_).^25^

**Figure 3:**
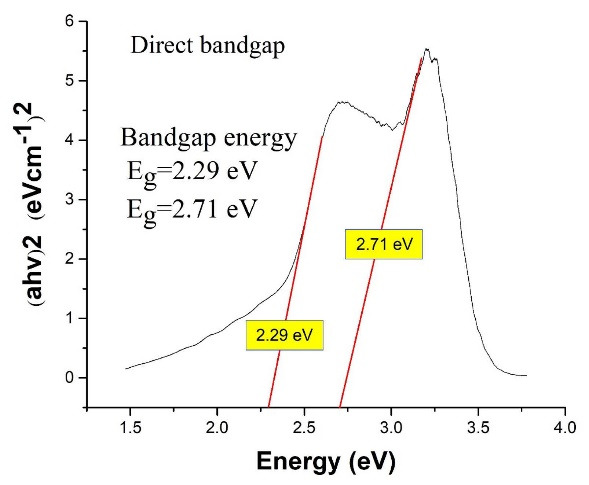
The plot of (αhν)2 vs. hν

 The composite’s bandgap was measured using the Tauc plot, as seen in Figure 3.

###  Photocatalytic activity, modeling and analyzing the results

 Experiments designed by the BBD method and their results are presented in Table 2. For example, in experiment No. 1, which was conducted at a relative humidity of 20%, an initial concentration of 3 ppm and a residence time of 30 minutes, the degradation efficiency of 20% was obtained for formaldehyde. The minimum and maximum degradation rates were 17% and 32%, respectively.

**Table 2:** Experimental design matrix and experimental results of the BBD

**Run** **No.**	**Factor**	**Factor**	**Factor**	**Response**
	**A**	**B**	**C**	**1**
	**Retention time**	**Relative humidity**	**Initial concentration**	**Percentage of formaldehyde degradation**
	**min**	**%**	**ppm**	**%**
1	30	20	3	22
2	90	20	3	29
3	30	60	3	19
4	90	60	3	26
5	30	40	2	24
6	90	40	2	32
7	30	40	4	17
8	90	40	4	24
9	60	20	2	28
10	60	60	2	25
11	60	20	4	23
12	60	60	4	21
13	60	40	3	25
14	60	40	3	25
15	60	40	3	25
16	60	40	3	24
17	60	40	3	24

 According to Adjusted R^2^ (0.9551) and Predicted R^2^ (0.9317) for linear model the software has selected the linear model to model operational factors’ effect on the photocatalytic degradation of formaldehyde.

 Analysis of variance (ANOVA) of regression parameters of the predicted response surface quadratic model for formaldehyde photocatalytic degradation efficiency is shown in Table 3. The model’s F-value and low probability value imply that the model is relevant for formaldehyde degradation. Since a *P* value less than 0.05 for each term indicates that this term has a significant effect on the response, all the main parameters of the model have a substantial effect on the formaldehyde photocatalytic degradation efficiency.^26^ But the terms related to the interaction between the parameters were not effective in the degradation efficiency due to having *P* value greater than 0.1. These terms were excluded from the study to improve the model.

**Table 3:** ANOVA table for linear model

**Source**	**Sum of squares**	**df**	**Mean square**	**F-value**	***P *** **value**	**Verdict**
Model	192.25	3	64.08	114.44	< 0.0001	Significant
A-Retention time	105.13	1	105.13	187.74	< 0.0001	
B-Relative humidity	15.12	1	15.12	27.01	0.0002	
C- Initial concentration of formaldehyde	72.00	1	72.00	128.58	< 0.0001	
Residual	7.28	13	0.5600			
Lack of fit	6.08	9	0.6755	2.25	0.2256	Not significant
Pure error	1.20	4	0.3000			
Cor total	199.53	16				

 Adequate precision greater than 4 suggests a desirable signal-to-noise ratio, which is 36.5034 for the proposed model in this study.^27^ The correlation coefficient of the present study for the impact of operational factors on formaldehyde degradation was 0.9635, which means that there is only a 3.65% probability of error in the model. The final regression model constructed from the coded factors will be according to the following equation:

Percentage of formaldehyde degradation = +24.29 + 3.63A−1.37B−3.00C

Eq. 2

 Examination of whether the selected model provides a good approximation of the results of actual experiments was performed using diagnostic charts such as predicted versus actual value plot and normal probability plot of the studentized residuals, and in this way, the adequacy of the model could be judged.

**Figure 4:**
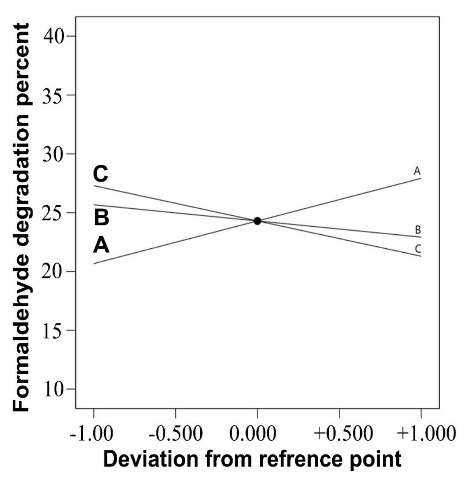
The process of changing degradation efficiency according to changes in operating factors

 The perturbation graphic illustrates the independent influence of each variable on formaldehyde degradation efficiency. As shown in Figure 4, a linear trend was observed in increasing the amount of degradation with increasing retention time. But, with increasing the initial concentration of formaldehyde and increasing the relative humidity, the degradation efficiency decreases. This decreasing trend has a greater slope with increasing the initial concentration of formaldehyde.

 The contour plots and 3D surface response of the model were utilized to assess the interactive relationships between independent variables and the response. By holding one variable constant and varying the other two in the test range, these graphs study the influence of those two metrics on the intended result.

 Using Design-Expert software version 12, operational parameters were optimized to achieve maximum degradation efficiency. According to the optimization steps in the software, to optimize the studied operational parameters, the amplitude of change in operational parameters for the software was defined within the test range. The target for formaldehyde degradation was defined as the maximum, and the degree of importance for this response was defined as 5. Also, the target for initial concentration, retention time, and relative humidity was defined as the maximum, minimum, and in range, respectively. The program generated ten operating conditions based on these characteristics, which are listed in the Table 4. As shown in Table 4, the removal of 24.06% formaldehyde is predicted under optimal operating conditions (relative humidity 20.00%, initial concentration 3.38 ppm, and retention time 56.23 minutes).

**Table 4:** Optimization results for formaldehyde maximum degradation efficiency

**Number**	**Retention time**	**Relative humidit**y	**Initial formaldehyde concentratio**n	**Percentage of formaldehyde degradation**	**Desirability**
1	55.49	20.00	3.39	23.95	0.55
2	55.43	20.00	3.38	23.97	0.55
3	55.27	20.00	3.39	23.92	0.55
4	54.92	20.00	3.39	23.89	0.55
5	55.10	20.00	3.37	23.98	0.55
6	55.28	20.00	3.41	23.87	0.55
7	56.23	20.00	3.38	24.06	0.55
8	56.52	20.00	3.40	24.06	0.55
9	54.99	20.00	3.35	24.02	0.55
10	54.21	20.00	3.39	23.80	0.55

## Discussion

 According to the definitions in the field of nanoparticles, they range in size between 10 and 500 nm, so according to the average size of the synthesized particles and the particle size distribution, synthesized Ag_3_PO_4_/TiO_2_ are in the nanoparticle range.^28^ The absorption spectrum of the Ag_3_PO_4_/TiO_2_ composite was a combination of the absorption spectra of these two components (TiO_2_ and Ag_3_PO_4_). This form of composite absorption spectrum indicates that there has been no chemical interaction between the two components, and the synthesized composite consists of two components TiO_2_ and Ag_3_PO_4_ which is consistent with previous studies in this field.^19^ Also given that the low Eg value indicates better absorption ability in visible light or natural sunlight, it can be concluded that the synthesized Ag_3_PO_4_/TiO_2_ composite in visible light has a better performance than any of its components.^14,29^

 A linear trend was observed in decreasing the amount of degradation with increasing relative humidity. One of the reasons for the decrease in formaldehyde removal efficiency by increasing the relative humidity can be the competition between formaldehyde molecules and water molecules for being adsorbed on the active sites of the photocatalyst.^20^ Although water molecules in the air are an essential source for producing hydroxyl radicals, which are then used to degrade formaldehyde molecules, relative humidity of 20% seems to be sufficient for this purpose, and higher values reduce the degradation efficiency.

 With increasing retention time, the degradation efficiency has increased. The reason for this increment could be the increased probability of formaldehyde molecules coming into contact with the photocatalyst and free radicals produced by the photocatalyst during longer residence time.^20^

 With increasing initial formaldehyde concentration, the degradation efficiency decreases due to the limited active sites of the photocatalyst, which is occupied by a limited number of contaminant molecules. *OH radicals, which are produced by the reaction of water molecules and photocatalyst active sites, play a very important role in the photocatalytic degradation process of formaldehyde because this substance oxidizes formaldehyde to carbon dioxide and water. As the concentration of formaldehyde increases, more pollutants cover the active sites of the photocatalyst. If the light intensity and irradiation time are constant, fewer photons reach the catalyst surface. As a result, less OH radicals are formed and subsequently the relative number of *OH radicals that attack formaldehyde also decreases. Therefore, an inhibitory effect is created in the photocatalytic degradation.^30^

 Typically, by increasing the initial concentration of the contaminant from zero to a certain concentration (from now on, referred to as M_max_), the degradation efficiency remains unchanged due to the lack of completion of the active sites of the catalyst by the contaminant. In this study, it seems that M_max_ is at concentrations below 2 ppm due to the high volume of the photoreactor.

 When the pollutant concentration increases, the pollutant around the photocatalyst creates a protective effect for other pollutant that prevents their destruction.^31^ In addition, an increase in the concentration of pollutants around the catalyst causes absorption and scattering of light, which, as a result, reduces the degradation efficiency of the catalyst and increases the competition for adsorption on the catalyst, which causes saturation of the catalyst.^32^

 In this study, due to existing limitations, a static reactor was used, but conducting experiments in dynamic reactors can bring the results closer to reality. To check the generalizability of the findings, it is recommended to conduct more studies with different pollutants and also different photocatalysts.

## Conclusion

 In this study, the effect of operational factors (initial formaldehyde concentration, relative humidity, and retention time) on the photocatalytic degradation efficiency of gaseous formaldehyde and the optimization of this process were investigated. The correlation coefficient of the model was 0.9635, which shows that the actual data are well consistent with the predicted data. According to the study, the photocatalytic degradation of pollutants from the air can be improved by optimizing the operational parameters effective in the degradation efficiency. The results of this study can be used in ventilation systems to remove pollutants in health care centers and industrial environments. In order to improve the performance of photocatalysts by optimizing the operating factors, it is suggested to study the effect of other operating factors such as light intensity.

## Competing Interests

 The authors have declared that there are no financial or nonfinancial competing interests.

## Ethical Approval

 The code of ethics was received from the ethics committee of Tabriz University of Medical Sciences with the specific ID IR.TBZMED.REC.1397.081.

## Funding

 This research study was funded by the Faculty of Health, Tabriz University of Medical Sciences.
